# Supplementary material for: The causal relationship between the human gut microbiota and pyogenic arthritis: a Mendelian randomization study
Source: Front Cell Infect Microbiol. 2024 Nov 26;14:1452480. doi: 10.3389/fcimb.2024.1452480 (PMC11629706; doi:10.3389/fcimb.2024.1452480)
Supplement: Supplementary file 1 [file DataSheet1.zip › mendelian test/ebi-a-GCST90027577.csv_forest.pdf]

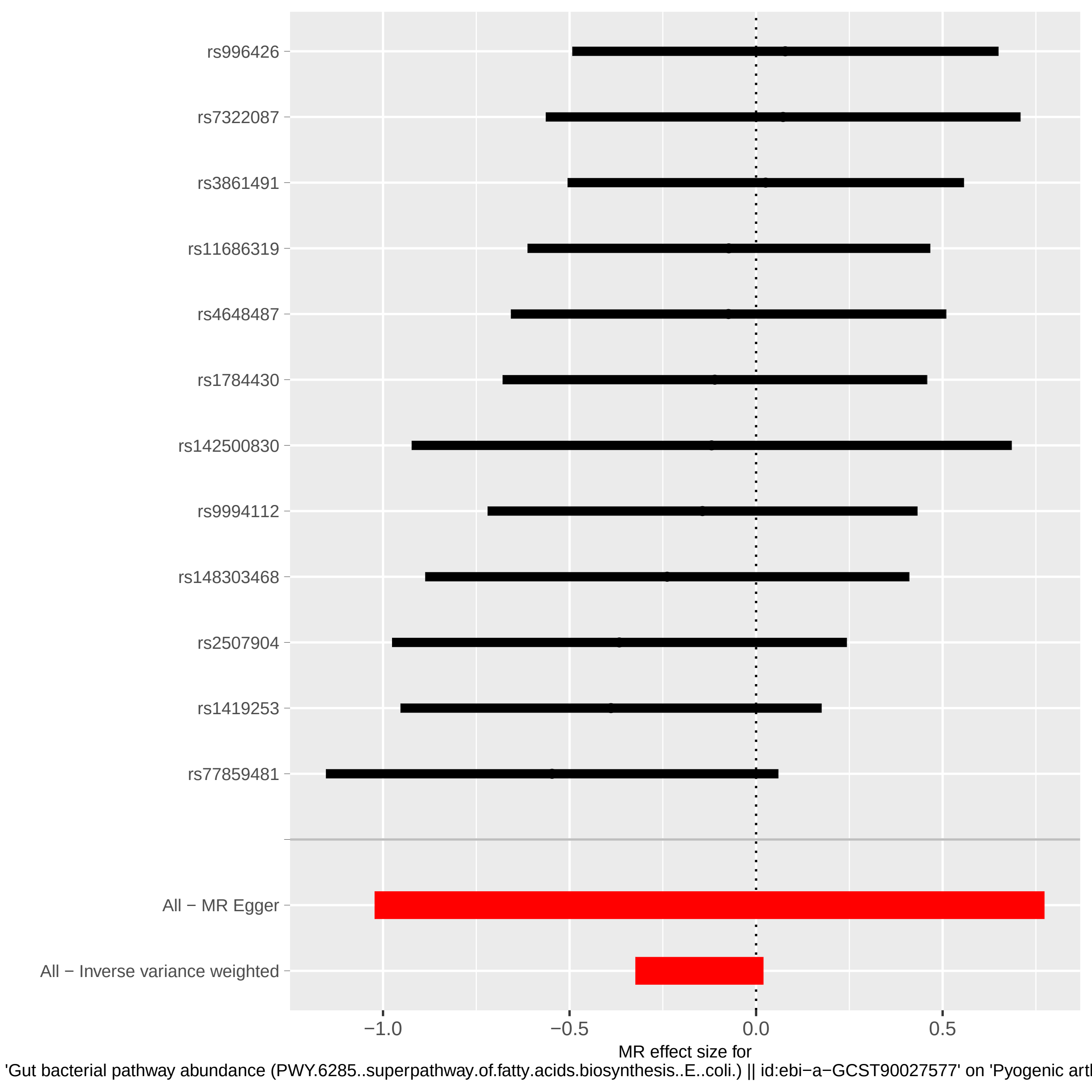
rs996426
rs7322087
rs3861491
rs11686319
rs4648487
rs1784430
rs142500830
rs9994112
rs148303468
rs2507904
rs1419253
rs77859481
All − MR Egger
All − Inverse variance weighted
−1.0
−0.5
0.0
0.5
MR effect size for
'Gut bacterial pathway abundance (PWY.6285..superpathway.of.fatty.acids.biosynthesis..E..coli.) || id:ebi−a−GCST90027577' on 'Pyogenic art
